# Towards Motor-Based Early Detection of Autism Red Flags: Enabling Technology and Exploratory Study Protocol

**DOI:** 10.3390/s21061971

**Published:** 2021-03-11

**Authors:** Mariasole Bondioli, Stefano Chessa, Antonio Narzisi, Susanna Pelagatti, Michele Zoncheddu

**Affiliations:** 1 Department of Computer Science, University of Pisa, Largo Pontecorvo 3, 56127 Pisa, Italy; mariasole.bondioli@gmail.com (M.B.); stefano.chessa@unipi.it (S.C.); susanna.pelagatti@unipi.it (S.P.); michele.zoncheddu@unipi.it (M.Z.); 2 Department of Child Psychiatry and Psychopharmacology, IRCCS Stella Maris Foundation, 56018 Pisa, Italy

**Keywords:** autism, internet of things, motor, inertial sensors, smart toys, children

## Abstract

Observing how children manipulate objects while they are playing can help detect possible autism spectrum disorders (ASD) at an early stage. For this purpose, specialists seek the so-called “red-flags” of motor signature of ASD for more precise diagnostic tests. However, a significant drawback to achieve this is that the observation of object manipulation by the child very often is not naturalistic, as it involves the physical presence of the specialist and is typically performed in hospitals. In this framework, we present a novel Internet of Things support in the form factory of a smart toy that can be used by specialists to perform indirect and non-invasive observations of the children in naturalistic conditions. While they play with the toy, children can be observed in their own environment and without the physical presence of the specialist. We also present the technical validation of the technology and the study protocol for the refinement of the diagnostic practice based on this technology.

## 1. Introduction

Autism Spectrum Disorder (ASD) is a severe multifactorial disorder characterized by an umbrella of specific peculiarities in the areas of the social communication, restricted interests, and repetitive behaviors [1]. The incidence of ASD is worldwide and recent epidemiological data estimated it to be higher than 1/100 [2,3,4]. Repetitive and restricted behaviors (RRB) that comprise repetitive and stereotyped movements, and restricted behaviors and interests are core behavioral traits of ASD.

As reported by Yu-Ching [5], children with ASD show significantly higher frequencies and degrees of repetitive and stereotyped movements with their bodies and the use of objects—turning around, jumping, swinging back and forth, tapping the head, flapping of hands, or spinning objects—than do toddlers who are TD (Typical Development) [6,7,8]. As indicated by Yu-Ching [5], the prevalence of overall motor deficits among people with ASD ranged from 50% to 80% with different impairments in basic gross and fine motor skills [9,10,11,12].

A recent meta-analysis of the motor literature in ASD revealed substantial motor coordination deficits pervasive across ASD diagnoses [13].

Sensorimotor timing and integration tend to be a persistent deficiency, as stated by Anzulewicz [14], although the essence of this disturbance and its impact on ASD as a sensory, motor and cognitive prediction condition requires further work to better elucidate it [15,16,17].

Nevertheless, motor timing and coordination disturbances can thwart the intentions of an individual [18]. As Anzulewicz [14] has suggested, prospective motor timing tests would tend to offer a means of testing young children for ASD if such motor markers could be identified.

Scientific studies, however, usually include optical motion monitoring, which is an expensive laboratory-based device requiring skilled technological activity. Clinical measurement of motor activity, on the other hand, is usually conducted by interpreter-coded surveys, such as M-ABC [19] or Mullen Scales [20], and the motor signature is not quantified precisely.

For clinical evaluation and research, more accessible and more reliable computational measures of motor performance are required. In particular, children at risk for ASD show less advanced abilities in grasping and object handling compared to infants who are TD in the first year of life [21,22,23].

Despite considerable evidence indicating delayed social communication and motor skills in ASD at-risk infants, few studies have looked at the movements of ASD infants in the sense of free-play exploration.

Kaur et al. [22] studied the manipulation skills of children with ASD using objects and found that infants at risk of ASD display a lower degree of object grasping and intentional falling than infants aged 6 to 15 months who are TD. In an object-sharing task, Srinivasan et al. [24] analyzed the movements of at-risk infants for ASD and found that at-risk walking infants display lower rates of giving objects to others, less approaches to caregivers, and lower step rates towards task-appropriate goals compared to those infants who are TD.

New technological advances have recently miniaturized inertial motion sensors, gyroscopes and magnetometers, and incorporated these into mobile consumer microelectronics, as documented by Anzulewicz [14]. In mobile phones, laptops, and in wearable devices such as smart watches and wristbands, they are now ubiquitous. These emerging technologies have, however, not yet been used to test motor function in children with ASD.

In this work, we present the development of the MoVEAS system, that makes use of a smart toy based on Internet of Things (IoT), machine learning and inertial motion sensors technologies.

MoVEAS can be used as a support to the specialist to assess the object manipulation behavior in children with ASD and TD through the automatized analysis of free-play movement performance in order to identify the motor signs of ASD. In particular, the ASD specialist can rely on the information about the type/quality of child play produced by MoVEAS to support his or her diagnostic decisions. Furthermore, still on the base of the information of MoVEAS, if the play session is video recorded, the specialist can rely on MoVEAS to find fragments of the video recordings that may deserve a deeper inspection, thus avoiding a lengthy, manual analysis. In our preliminary works, we presented the hardware and the basic data fusion algorithm [25,26,27] of MoVEAS, its preliminary validation conducted with two children and aimed at testing the hardware and the communication and data collection procedure in real conditions [25]. Here, we present the final version of the system that includes the full activity recognition stack validated in laboratory and that it is ready for an experimental pilot. Specifically, the activity recognition component of MoVEAS has been validated by means of a dataset of movement patterns created in laboratory, representative of several possible movements that can be applied to the toy during play.

The rest of the paper is organized as follows: Section 2 reports the state of the art concerning the combination of IoT and artificial intelligence to recognize human activities, Section 3 and Section 4 introduce the MoVEAS system and its validation, respectively, and Section 5 presents the protocol for the pilot study. Finally, Section 6 draws the conclusions.

## 2. State of the Art

In recent years there has been a growing interest in pervasive technologies and sensor systems aimed at refining the detection of typical movements and characteristic features of individuals with ASD [28]. The observation, both for diagnosis and for therapeutic intentions, of specific behaviors of children with ASD is increasingly inclined to exploit the contributions of non-invasive IoT solutions, to monitoring and collecting even more accurate data in this regard [28,29,30,31]. In this perspective, Goodwin et al. [28] investigated with their work the potential of the automatic detection and classification of stereotypical movements in six children with ASD using three wireless accelerometers (in both the wrists and on the chest). They trained their system with the purpose of detecting repetitive behaviors, achieving good results. To this purpose they produced a dataset of accelerometer data obtained in experimental sessions, conducted in different environments, specifically aimed at stimulating stereotypical behaviors.

In other studies, the recognition of stereotypical movement approaches has exploited Kinect-based research [30,31]. In particular, in Kang et al. [30], the recording of Kinect camera (used to film 12 actors performing three separate stereotypical motor movements each) is exploited to recognize these movements using two different approaches using the Visual Gesture Builder (VGB) and Matlab. VGB is an interactive tool for building models of body gestures using a machine learning classifier. VGB leads to the best results and the true positive detections were above 90% for the three gestures taken into account (flapping, spinning and body rocking). Looking to the wider field of the human activity recognition, there are a large number of papers, not focused on the ASD diagnosis or monitoring, that base their research results about motions’ detection and classification on the combination of the triad of sensors exploited in our paper (accelerometer, gyroscope, magnetometer) and different deep learning approaches [32,33,34,35,36,37,38,39].

Among them, for example, the work of Najafi et al. [32] presents a new method of physical activity monitoring based on a 2D accelerometer and a gyroscope fused in a sensor system to wear on the chest. The device used is able to detect body postures (sitting, standing, and lying) and periods of walking in elderly persons using only one kinematic sensor also to be kept on the chest. We highlight a wide number of human activity recognition research based on a single accelerometer system, exploited to pursue extremely different aims. With accelerometer-based systems, for example, the researchers as that of Sekine et al. [35] attempt to distinguish walking on level ground from walking on a stairway, or to give the input to algorithm for analyzing and classifying human activity (as standing, sitting down with lowering subjects head, sitting down and leaning against, lying down straight, lying upside down, walking, going up/down stairs, running) using a body-fixed triaxial accelerometer on the back, as in the case of Lee et al. [37] or on the wrist as in Panwar et al. [39].

Although the methodology adopted in our work has some elements in common with the works presented so far, the objective of our work differs significantly as we do not aim to classify postures or specific movements (e.g., a movement of a wrist), but, rather, to classify complex movements applied to a toy by a child (e.g., making the toy fly). This classification is useful as it provides automatic information about the type/quality of activities in the free play of a child that, in turn, can be used by the specialist in the diagnosis of ASD.

## 3. The MoVEAS System for Motion Capture

### 3.1. Reference Scenario

Observing children while they play is a common test adopted by specialists to identify ASD at an early stage. However, in practice the observation is carried out at the clinic with standard sets of toys. This setting for observation is not ideal because the environment and the toys used are totally new for the child. The main idea of MoVEAS is to develop and use a “smart” toy that embeds sensors in the standard evaluation session in clinic, in order to collect data that may contribute to improve and to make more objective observations of the child’s play by the specialist. In the future, we expect that MoVEAS may enable monitoring of the play also in other contexts, like at school or even at home, to monitor how play changes when toys become more familiar for the child. MoVEAS smart toys are designed to enable easy and simple use in the conventional sessions of therapy conducted in clinics. Their purpose is to allow specialists to develop new, non-invasive diagnostic protocols for ASD, that take into account the continuous data produced by the MoVEAS smart toy about the playing movements of the child under observation. Note that the toy is not meant to be used alone, but rather in combination with the traditional observation of the child conducted by the therapist during the clinical process of diagnosis. Another possibility, however, is to use the toy remotely, but still under the therapist’s control, which would give to the child the possibility of behaving more freely and without other people around.

Keeping in mind these use cases, the system is designed to manage all set up phases of the system (association of toy/child, management of the users, etc.), interaction between the child and the toy, the centralized sensor data collection in the MoVEAS server and its analysis, and the final representation of the results of the analysis by means of the dashboard for the clinician. Concerning normal use sessions, the clinician can control and initiate the following steps: (1) the clinician uses a standard browser to access the MoVEAS dashboard using his/her credentials; (2) starts a new play session about a patient and the connected smart toy, along with the data collection procedure; (3) the smart toy in use in the session automatically provides all the sensors readings (that are thus related to the specific patient activities during the play session) to the server, which stores them in its database; (4) the MoVEAS component of activity recognition automatically analyses the sensor data to provide the sequence of recognized movements applied to the toy; (5) the result of the analysis is stored and also displayed on the clinician dashboard. Through the application User Interface, the clinician can also add new patients and toys to the database, manage a capture session, visualize the recording of a given session and analyze the detected movements.

### 3.2. Smart Toy Prototype Overview

MoVEAS consists of the following components: the smart toy, the activity recognition component, the backend and data storage and the user interface [25,26,27].

#### 3.2.1. Smart Toys

**Figure 1:**
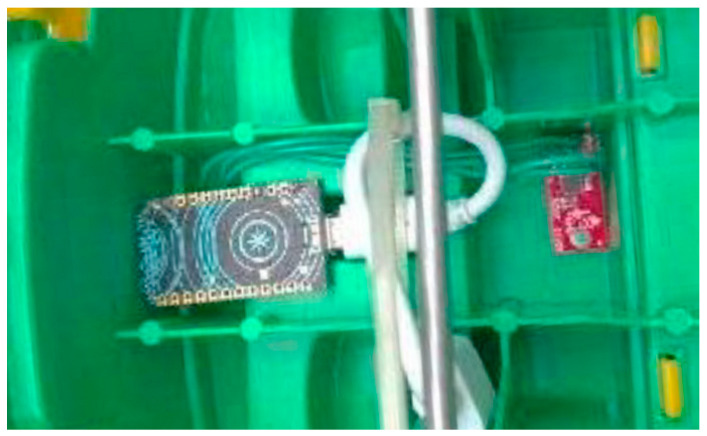
Detail of a sensor installation in a toy truck.

**Figure 2:**
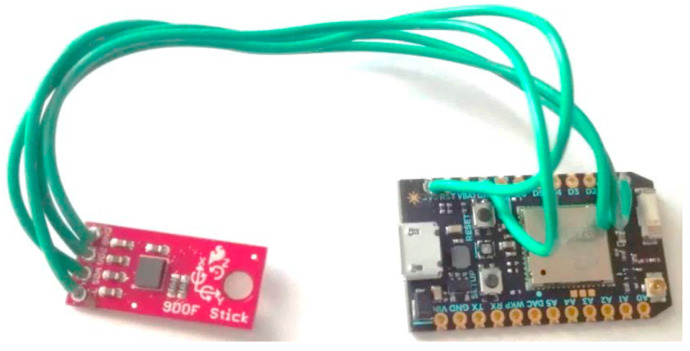
The Particle Photon with the accelerometer.

The smart toy is aimed at sensing the force and the movement direction applied to it by a child, and at measuring other data about the play, such as how long the toy is used by the child and when. It achieves these objectives by inertial sensors that are embedded within the toy itself, so as to avoid being invasive. In our case, we embedded a three-axes accelerometer, a gyroscope and magnetometer transducers in the toy. Note, however, that the raw data obtained by these sensors alone is not sufficient to obtain directly the force and direction of movement. This is because the accelerometer transducer produces noisy data that is also affected by the Earth’s gravity, and the gyroscope is affected by drifts. We solved these issues by a two-step activity recognition algorithm that implements a first step of data fusion and a second step of data classification-based machine learning. We implemented the current sensor prototype over Particle Photon, an Arduino platform, and the server over a Raspberry PI device. Figure 1 shows the sensor installation in a truck toy and Figure 2 shows the naked device with the acceleration sensor.

At the current state of our project, we implemented two smart toy prototypes using two toys for children which are part of the Autism Diagnostic Observation Schedule (ADOS), which is the gold standard tool for the evaluation of ASD: an airplane and a truck. For the study protocol, we also used other toys such as dolls, toy tools for the kitchen and stuffed animals. In the literature, it is reported that girls and boys with ASD are largely equivalent in their play complexity. However, despite similar play, girls and boys with ASD differ in a number of ways in their toy engagement, replicating traditional gender differences [40].

**Figure 3:**
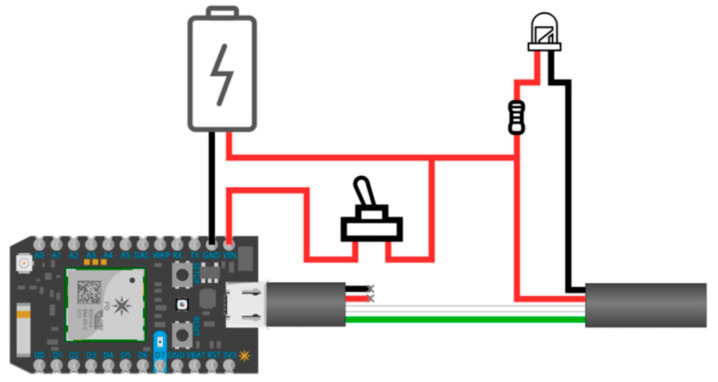
The circuit for battery charging.

These two smart toys have been configured with the MoVEAS system (which, however, can support a large number of smart toys). For the purpose of interaction between the smart toy(s) and the MoVEAS server, we adopted MQTT, a well-known IoT protocol. During the project, we experimented with two power supply methods for the smart toys. The first method used a powerbank of 2600 mAh, which was rechargeable using an USB port. However, even though the powerbank was relatively small sized (31 × 22.5 × 73 mm), it was still significantly larger than the other electronic components and was relatively heavy, which was a problem especially for the Super Wings. Moreover, to switch the toy on and off would require plugging and unplugging the powerbank from the USB port. Thus, we experimented an alternative supply using a small 500 mAh rechargeable lithium polymer battery connected to the device through its GND and VIN pins, small switch, a micro-USB extender and a custom circuit (Figure 3).

**Figure 4:**
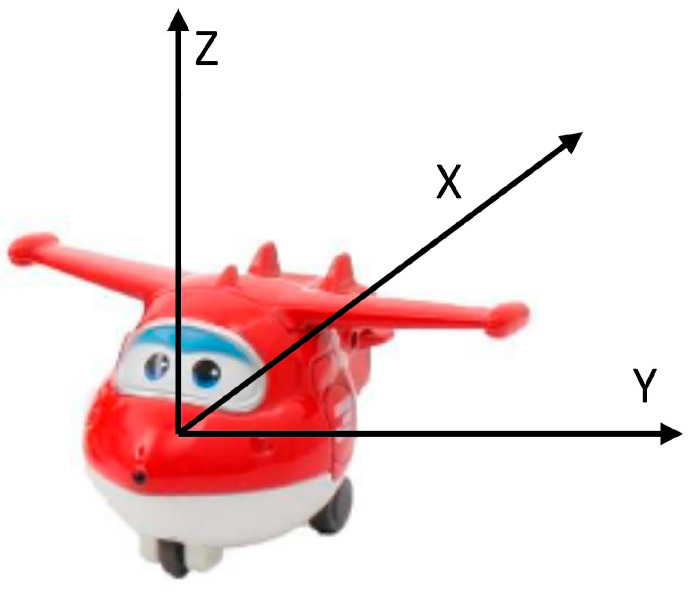
Super Wings smart toy: a detail of the orientation of the accelerometer axes.

The sensor was placed within the toy to align the axes of the accelerometer with those of the toy. For example, in the case of trucks, the X-axis of the accelerometer is parallel to the sliding direction of the wheels and the Y-axis is transversal. Figure 4 shows the orientation in the case of the airplane.

#### 3.2.2. Activity Recognition Component

**Figure 5:**
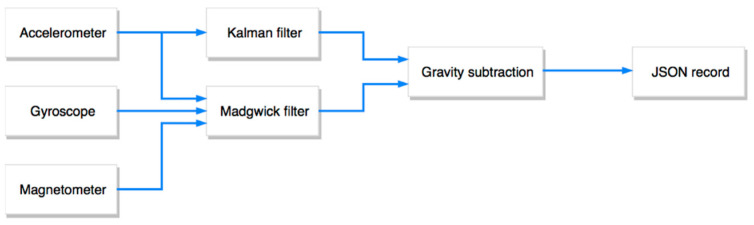
The data fusion algorithm.

The activity recognition component operates in two steps. The first step is a data fusion algorithm (Figure 5) that processes the time series consisting of the tuples of raw accelerometer, gyroscope and magnetometer data. The output of the data fusion algorithm is the time series containing tuples of movement direction and intensity of the toy and its orientation, where each output tuple is produced for each new tuple in input. The output time series and the raw data time series are inputs to the second step (classification) based on neural networks that classify them as recognized movement of the toy.

For what concerns the data fusion algorithm, note that a correct estimation of the orientation and movement is a rather complex task. Each single input data, either from the accelerometer, the gyroscope or the magnetometer, was insufficient to obtain orientation or movement direction data. This is because of the noise in the transducer readings, but also because of the Earth’s gravity and drifts. To deal with drifts of the gyroscope, we also used a magnetometer (whose measurements are not affected by drifts as they refer to the Earth’s magnetic field). We implemented the Madgwick algorithm [39], which provides a quaternion representation of the orientation of the toy. For the movement direction and intensity, we first evaluated the contribution of the Earth’s gravity along the three axes on the base of the toy orientation that resulted from the Madgwick algorithm, and we then subtracted this contribution from the raw accelerometer data. This algorithm was made stable by means of a Kalman filter. The acceleration was given in a global reference system, so it was rotated by the angles given by the orientation to align it to a local reference system (the toy itself). Each input and output tuple of the data fusion algorithm was stored in a single JSON record, that also includes the filtered acceleration data, and the toy orientation in different forms: in quaternion representation, in Euclidean angles (roll, pitch and yaw), and in spherical coordinates.

The second step consisted of the classification of the movements of the toy by analyzing the time series patterns of raw and pre-processed data. This step classified the movements applied to the toy into six classes: forward (the toy is pushed forward), backward (the toy is pushed backward), walking (the child walks while keeping the toy in his hands), throw, flight (the child moves the toy to make it “fly”), plus, of course, the case in which the toy is unused and hence still. Since not all patterns had the same recording time (for example, the duration of the movement of the toy pushed forward is much smaller than the one where the toy is thrown against a pillow–where the toy is thrown by the hand first, then flies in the air and finally hits the pillow), the goal of this second step of classification was to classify the movements of the toy under different variable length sequences. We considered two different neural network models for the implementation of the classifier: the time delay neural network (TDNN), chosen for its simplicity among the shift-invariant models, and the recurrent neural network (RNN), chosen also for its ability to recognize sequences of data of variable length. The two models have been configured, trained, and validated by means of a dataset collected in laboratory. The details of these steps are in Section 4.

#### 3.2.3. Backend and Data Storage

Each single output of the data fusion algorithm was sent to the MoVEAS server in a JSON record within an MQTT message (by means of the Mosca broker) over a Wi-Fi connection. In turn, the server stores the JSON record in its database. In further developments, we plan to add an external storage module to allow offline data recording, and also to embed the neural network model on the device itself, to store the already labeled data instead of the sensors’ data, saving storage space.

#### 3.2.4. MoVEAS User Interface

**Figure 6:**
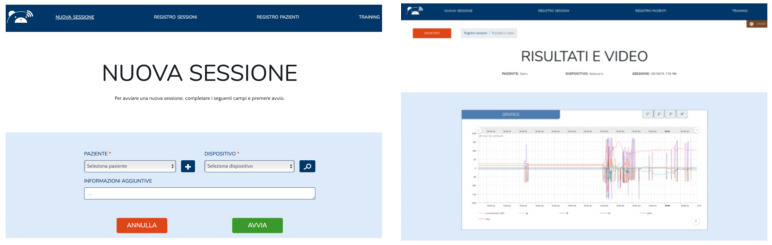
Starting a new session (**left**), showing raw data for a session (**right**).

**Figure 7:**
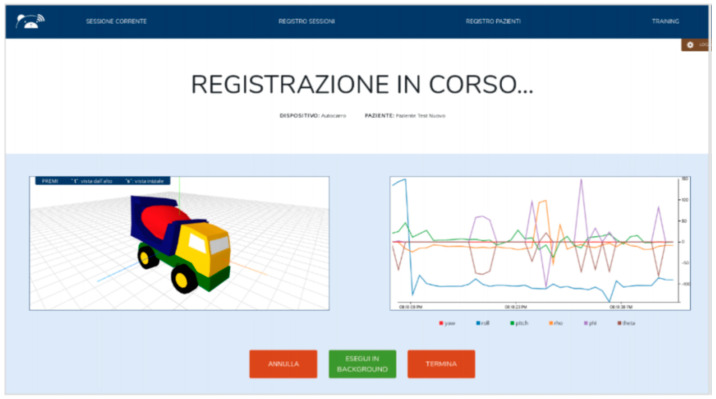
Monitoring a recording session.

The interaction of the clinical staff with the system was supported by a web application (Figure 6 and Figure 7). The user interface is currently in Italian and can be used on a mobile device or PC. Figure 6 (left) shows how a new session can be started by selecting the patient (“PAZIENTE”) from a drop-down menu, including all the patients already registered in the system. In the same way, a device/toy (“DISPOSITIVO”) can be selected from the right drop-down menu. The clinician can add information using the “INFORMAZIONI AGGIUNTIVE” field below. The session can be started or clicking on the “AVVIA” button or undone clicking on the “ANNULLA” one. The menu at the top of the screen allows the user to quickly jump to the pages related to the patient register (“REGISTRO PAZIENTI”), to the log of the previous sessions (“REGISTRO SESSIONI”) and to the training (“TRAINING”). We can also add a new patient (button marked with a “+”) or search for a session (button marked with a magnifying glass). Figure 6 (right) shows the raw data collected during a session. Through this interface the clinician can access all the data collected in the session (“RISULTATI”) and the video recorded (“VIDEO”). During a session, a clinician can monitor the movements of a toy and the data collected in real time. Figure 7 shows a monitoring interface (“REGISTRAZIONE IN CORSO” means “recording in progress”). In this case, the toy movements on the left-hand side are synchronized with the data collected on the right-hand side. Clicking on the buttons on the lower part of the interface in the session can be undone (“ANNULLA”), ended (“TERMINA”) or executed without monitoring (“ESEGUI IN BACKGROUND”).

## 4. Technical Validation

### 4.1. Dataset Collection

To train and validate the activity recognition models, we collected a dataset in laboratory. This dataset was produced by a researcher using the toy and the user interface in a specific modality for data acquisition, which also supports labelling of recorded patterns. The dataset consists of 1240 patterns:

- 120 patterns with the toy moving forward;
- 120 patterns of the toy moving *backward*;
- 250 patterns of simulated *flight*;
- 250 patterns with the toy *still*;
- 250 patterns of the toy carried while *walking*;
- 250 patterns of the toy *thrown* (against a pillow).

Each pattern is a tuple that contains all the output of the data fusion algorithm plus the raw data from the accelerometer, gyroscope and magnetometer, produced at a frequency of 22 Hz. The optimal pattern length for these six movements was found to be 14 samples, hence each pattern is 636 milliseconds long.

The dataset was divided into the training and testing portions (respectively 83% and 17% for the *forward* and *backward* patterns, 80% and 20% for the others), the training portion was then divided into the training and validation ones, with a split of 60% and 40%, respectively, and the whole training/validation portion was shuffled before every training.

### 4.2. Neural Network Models Training and Validation

As discussed in the previous section, we considered two neural network models, namely TDNN and RNN, to implement the classification of the movements of the toy. Here we discuss the training and validation process for both models. For what concerns the overfitting detection, this was based on the accuracy on the validation set; when the increment of accuracy on training did not match an increment on validation, the model was discarded. For both the TDNN and RNN, the model with the highest validation accuracy was chosen. More precisely, the model selection for the TDNN was performed with a grid search on the number of filters and size of the kernel, while for the RNN the main hyperparameter was the number of units of the single recurrent layer.

In TDNN, model selection started from the simplest topology: an input layer with data normalization, a convolutional hidden layer with a ReLU activation function (that performs better than tanh), a max-pooling layer (that performs better than the average-pooling one), and an output layer for the pattern classification with a softmax function, to obtain values between 0 and 1 that represent the probability of the classified pattern to belong to each possible class.

We considered six features for the training: the gravity-free acceleration values in the three axes and the gyroscope raw data; the gyroscope data was used to discriminate some cases that are not well distinguished only by the acceleration. Some tests quickly showed that adding more features, like orientation, velocity data, or raw sensors data, made the network generally harder to train, and most often gave no advantage at all in the overall accuracy.

The search for the two most important hyperparameters, the kernel size and the number of filters, was performed through a grid search, as shown in Table 1. For each table’s entry, the network was tested 10 times, with shuffled data between testing and validation set for each run. Outliers were omitted from the average.

The values 9 and 7 were optimal for the number of filters and the kernel size, respectively, as they gave the best result in the validation set, and in particular:

- the number of filters up to 7 and a kernel size up to 5 made the network able to classify correctly only after a long and unstable training;
- from 7 filters upwards, the training curve was stable, and the overfitting occurs only from 15;
- kernel sizes from 9 upwards tended to overfit the network; the patterns were initially made of 22 samples, and the sliding window results were too big;
- with filters bigger than 20, the overfitting was mitigable only with very low kernel sizes, but in that case the network was hard to train, and very easy to underfit.

More complex topologies were tested but adding hidden layers only led the neural network to overfitting. The best training curve was obtained using a 16-patterns long mini-batch.

**Figure 8:**
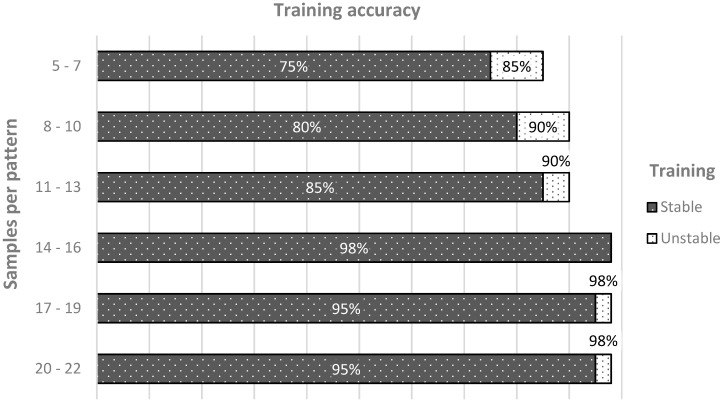
Training accuracy of TDNN with patterns of different lengths (from 5 to 22).

The next step was to achieve the maximum accuracy with the least possible amount of data. For this purpose, the network is trained several times with different pattern lengths (Figure 8) and the best choice for a stable and accurate training resulted in 14 samples per pattern, with the 22 Hertz sampling meaning 636 milliseconds long patterns. Up to 11–13 samples, the network was not able to achieve good performance in training, being hard to train and from underfitting, while from 17 samples and more, the network fit the noise, and was overfitted. The convolutional layer’s weights were initialized randomly in the uniform range [−0.000001, 0.000001]. To keep the network complexity under control, a weight decay approach was used, with tested values in the range of [0.001, 0.05]. The optimization algorithm chosen for the learning was Adam because of its efficiency [41].

Concerning the RNN, the topology was similar to that used for the TDNN: the input passed through a normalization layer before going to the recurrent layer, and the activation function was ReLU for all layers, except for the last one, that used the softmax function, particularly suitable for classification. By using fixed-length patterns, however, we were not exploiting the full potential of the RNN that’s capable of recognizing variable-length sequences. This feature can be exploited in future work with a custom dataset for this model.

The chosen topology, which ensured good fitting without instabilities during the training, consisted of one recurrent layer and two densely connected layers, with two dropout layers before them. In particular, the number of nodes in the recurrent layer was crucial for the model selection:

- up to 5 units, the network was not able learn the training samples nor to generalize;
- up to 10 units, the validation accuracy reached 90%, but when the training continued, it overfit;
- up to 12 units, the network was hard to train, and with 14 units the network was trained smoothly and the validation accuracy grew up to 93.5%;
- from 16 upwards, stricter regularization was needed, but the dropout layers make the training stable and avoid overfitting. In the end, the best number of units resulted to be 20.

The explored range of dropout for both layers was [0, 0.5], and the network was finally trained with a mini-batch size of 16 patterns.

Adding features besides the gravity-free acceleration and the gyroscope data gave most often no advantage at all, and sometimes made the training harder and unstable, also with more layers and more recurrent units. In RNN, reducing the number of samples did not improve the validation accuracy as for the TDNN, but the performance significantly decreased only for less than 14 samples per pattern, accordingly to the results achieved with the TDNN.

### 4.3. Results of Classification

**Figure 9:**
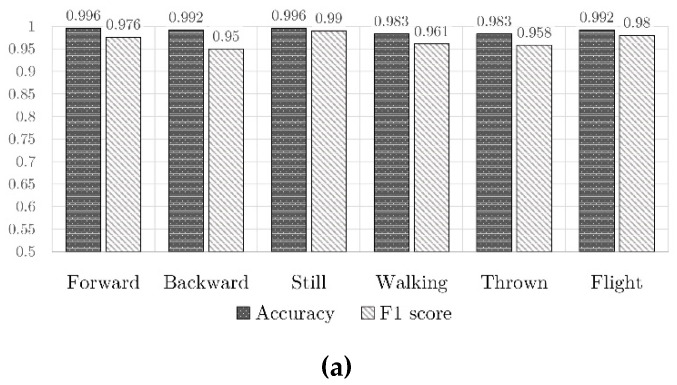

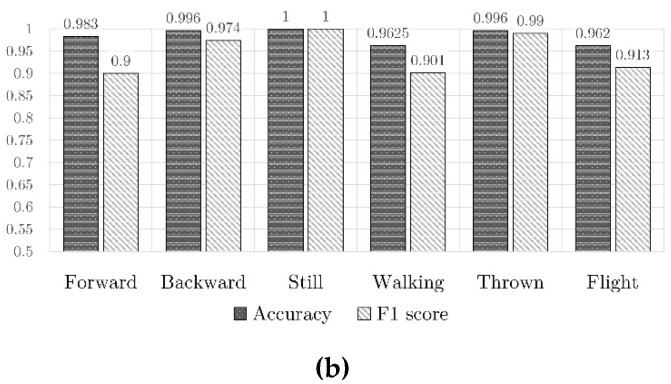
Accuracy and F1 score for (**a**) TDNN (**b**) RNN.

The results in terms of accuracy and F1 score for both TDNN and RNN are shown in Figure 9. It is seen that TDNN provided a better validation accuracy and a smaller loss value. Moreover, in almost every test, TDNN showed smoother training and validation curves, and was easier to train effectively. Note however that the high accuracy obtained in validation and testing might not reflect the real network’s performances in analyzing real play sessions, in fact, in validation and testing the patterns have a well-defined start and end, while in a play session there is a continuous stream of data. For the classification, the network was given a fixed length subsection of the stream for every twenty-second of a second, then the subsection’s bounds slid forward, like a sliding window, through the whole stream. When the subsection contained only a single pattern, the networks behaved exactly like in the validation/testing scenario, but when there was an overlap between two patterns (e.g., the toy stops flying and stays still), the classification was harder as it’s not a case that the network was trained to deal with. For this reason, we performed a further experiment with data from a 46-second-long session of simulated play. The network was able to accurately recognize the still, forward, and backward patterns while in flight and especially while walking, the overall classification was correct, with some very short misclassifications. We plan to investigate this issue in detail with real play session recordings.

## 5. Study Protocol

### 5.1. Participants

We enrolled 50 preschoolers, 25 with idiopathic ASD and 25 with Typical Development (TD) who were recruited from Autismo Pisa (APS) and local kindergartens sited in Pisa in order to conduct an exploratory study. All children recruited were assessed from experts in ASD.

The inclusion criteria for ASD were (a) age 3–5 years, (b) diagnosis of ASD according to Diagnostic and Statistical Manual-5th edition (DSM-5) [1], meeting criteria for ASD on the Autism Diagnostic Observation Schedule-2nd Edition (ADOS-2) [42], and (b) nonverbal developmental level > 70 on the Griffiths Mental Developmental Scales (GMDS-III). The exclusion criteria were (1) severe sensory impairments (i.e., hearing or visual); and (2) an identified genetic disorder that would impact on ability to participate or affect validity of data.

Regarding the TD sample, they required a scoring of <20 on the CARS-2 [43]; a T score < 60 at Withdrawn and Pervasive Developmental Problems (PDP) for the Child Behavior Check List 1,5–5 [44]; and no siblings or family history of ASD or other neurodevelopmental disorders.

### 5.2. Measures

Standardized protocols were used in order to assess ASD and TD samples.

The ADOS-2 [42] is a semi-structured, standardized assessment of communication, social interaction, play and restricted and repetitive behaviors. It provides a highly accurate picture of current symptoms, unaffected by language. It can be used to evaluate almost anyone suspected of having ASD from 1-year-olds with no speech, to adults who are verbally fluent.

The Peabody Developmental Motor Scales Second Edition (PDMS-2) [45] combines in-depth assessment with training or remediation of gross and fine motor skills of children from birth through 5 years.

The Childhood Autism Rating Scale, Second Edition (CARS-2) [43] helps to identify children with ASD and determine symptom severity through quantifiable ratings based on direct observation. Widely used and empirically validated, CARS-2 has proven to be especially effective in (a) identifying children with ASD and those with severe cognitive deficits, and (b) distinguishing mild-to-moderate from severe autism. CARS 2 addresses the following functional areas, among others: (1) relating to people; (2) body use; (3) visual response; (4) listening response; (5) taste, smell, and touch response and use; (6) verbal communication; (7) nonverbal communication; and (8) level and consistency of intellectual response.

The GMDS-III [46] is a standardized developmental test for children from birth to 96 months of age. It comprises six scales, but because of our aims only two subscales was administered: eye and hand coordination, and performance. Raw scores were computed for each subscale and converted to general quotient scores, using tables of the analysis manual.

Repetitive Behavior Scales-Revised (RBS-R) [47] is a 26-point parent questionnaire for assessing repetitive behaviors in children with ASD.

Sensory Processing Measure-Preschool (SPM-P) [48] requires just 15 to 20 min, the Home and Main Classroom Forms yield eight parallel standard scores: social participation, vision, hearing, touch, body awareness (proprioception), balance and motion (vestibular function), planning and ideas (praxis), and total sensory systems. Scores for each scale fall into one of three interpretive ranges: typical, some problems or definite dysfunction.

The Vineland Adaptive Behavior Scales, Second Edition (VABS-II) [49,50], was administered as a parent interview and was used to assess the ability of children to perform the daily activities required for personal and social sufficiency. The VABS-II uses four specific domains: communication, daily living skills, socialization, and motor skills. For our aims only motor skills will be evaluated.

### 5.3. Procedures

Research and clinical staff confirmed eligibility and obtained informant consent. Each case was assigned a participant identification (ID) number. All MoVEAS sessions were videotaped and performed on a different day than those used for testing children. The behavioral evaluations (see Measures section) were performed by experienced clinicians and by parents during the research project.

Regarding MoVEAS, therapists were asked to carry out imitation tasks with children using sensorized toys.

The imitation tasks were video recorded in order to do a qualitative and quantitative analysis. The aim of this video was two-fold: (a) to examine the functional and symbolic use of objects and the presence of restricted and repetitive motor behaviors during the use of toys; and (b) to correlate the qualitative data with the objective data as captured by MoVEAS (accelerometer, magnetometer and the gyroscope data).

### 5.4. Data and Statistical Analysis

All data was collected in order to:

(a) compare ASD vs. TD groups regarding a part of the clinical standardized protocols (SPM-P; RBS-R; CBCL; CARS);
(b) compare ASD vs. TD groups regarding object manipulation as captured by MoVEAS;
(c) correlate, in ASD and TD groups, MoVEAS data with qualitative data;
(d) correlate, in the ASD group, the scores of clinical standardized protocols with the data of the object manipulation captured through MoVEAS.

## 6. Conclusions

The MoVEAS project was carried out over the past two years with the final goal of exploiting the potential of recent technological developments; specifically, to integrate sensors into commonly used toys to get new insights in motor abilities of ASD children when observed in a natural environment.

In particular, we integrated a Particle Photon in a “Super Wings” airplane and in a toy truck, building up a system able to record data from play sessions and analyze them using neural networks, to recognize the movement while the child was playing. We considered two neural network models, TDNN and RNN, to implement the classification of the movements of the toy and analyzed their performance through a training session. TDNN provided a better validation accuracy and a smaller loss value in all trials. Moreover, in almost every test, the network was able to accurately recognize the still, forward, and backward patterns, while in flight and especially walking, the overall classification was correct, with some very short misclassifications.

Despite the good results achieved in this experimental phase, only working with children with ASD can reveal the actual project’s usefulness. For this reason, we planned a pilot study to be conducted with children with ASD and TD, whose result will be the final refinement and validation of the machine learning model with data obtained from real uses of the toy by the children, and the validation of the usability and acceptability of the interfaces and of the protocol in general. In this perspective, we discussed the study protocol of our forthcoming test including a sample of already diagnosed ASD children and a control group of neurotypical children. This study also has the objective of devising and assessing a suitable diagnostic protocol that may make use of MoVEAS. A potential future direction is to sensorize several toys, to be included in the ADOS-2, which is the gold standard tool for evaluating children with ASD. In the long term, we expect to frame MoVEAS into an experimental medical protocol to be used first in hospitals in the region Tuscany, and then, possibly, to other hospitals nationwide.

The study also gives the opportunity to improve the system performance and to investigate its behavior. In the future, we would investigate how to “flatten” the small spikes that affect the continuity of a classification, in order to obtain compact chunks to highlight with different colors in the session’s progress bar. Another path to be explored is to train the recurrent neural network with a variable-length patterns training set, and to find the best way to choose the length of the session’s subsequence dynamically, in order to exploit the full network’s capabilities.

## Figures and Tables

**Table 1:** Average training accuracy for the time delay neural network (TDNN).

		Kernel Size			
		3	5	7	9
Filters	3	90.00%	92.57%	95.00%	98.25%
	5	94.50%	95.50%	98.50%	99.00%
	7	98.80%	99.00%	99.87%	99.90%
	9	97.93%	99.00%	99.65%	99.87%
	11	98.06%	98.52%	99.59%	99.83%
	13	96.94%	99.53%	99.91%	99.84%
	15	99.20%	99.88%	99.90%	99.90%

## Data Availability

The data presented in this study are available on request from the corresponding author.
